# K27Q/K29Q mutations in sphingosine kinase 1 attenuate high-fat diet induced obesity and altered glucose homeostasis in mice

**DOI:** 10.1038/s41598-020-77096-w

**Published:** 2020-11-18

**Authors:** Jing Xie, Yong Shao, Jin Liu, Meilan Cui, Xiuxiao Xiao, Jingbo Gong, Binghua Xue, Qunwei Zhang, Xianwen Hu, Haifeng Duan

**Affiliations:** 1 Key Laboratory of Experimental Hematology, Beijing Institute of Radiation Medicine (BIRM), No. 27, Taiping Road, Haidian District, Beijing, 100850 China; 2 Laboratory of Cell Engineering, Beijing Institute of Biotechnology (BIB), No. 20, Dongdajie Street, Fengtai District, Beijing, 100071 China

**Keywords:** Acetylation, Diabetes, Metabolic syndrome, Obesity

## Abstract

Obesity and its associated metabolic disorders are increasingly impacting public health worldwide. Sphingosine kinase 1 (Sphk1) is a critical enzyme in sphingolipid metabolism that has been implicated in various metabolic syndromes. In this study, we developed a mouse model constitutively expressing pseudoacetylated mouse Sphk1 (QSPHK1) to study its role in regulating glucose and lipid metabolism. The results showed that QSPHK1 mice gained less body weight than wide type (WT) mice on a high-fat diet, and QSPHK1 mice had improved glucolipid metabolism and insulin. Moreover, QSPHK1 mice had alleviated hepatic triglyceride accumulation and had high-fat-diet-induced hepatic steatosis that occurred as a result of reduced lipogenesis and enhanced fatty acid oxidation, which were mediated by the AMPK/ACC axis and the FGF21/adiponectin axis. Collectively, this study provided evidence that the K27Q/K29Q mutations of Sphk1 could have a protective role in preventing obesity and the related metabolic diseases. Hence, our results contribute to further understanding of the biological functions of Sphk1, which has great pharmaceutical implications.

## Introduction

Obesity is a multifactorial disease that represents a growing threat to public health worldwide. It was estimated that nearly a third of the world’s population is overweight or obese^1^. Obesity is closely associated with type 2 diabetes and cardiovascular disease, and the basis for this link is that obesity contributes to the development of insulin resistance, which is characterized by the inefficient action of insulin to inhibit the output of liver glucose and promote peripheral glucose uptake and utilization^2^. Moreover, obesity is considered as a risk factor for many other diseases, including nonalcoholic fatty liver disease (NAFLD), and several cancers^3,4^.

Sphingosine (Sph) and its metabolite sphingosine-1-phosphate (S1P) are important signaling molecules that regulate many biological processes, including differentiation, cell survival and apoptosis, angiogenesis, and inflammation^5,6^. S1P is formed by phosphorylation of Sph catalyzed by sphingosine kinases (Sphks), which plays central roles in sphingolipid metabolism^5^. Sphks are evolutionarily conserved and there are two mammalian isoforms (i.e. Sphk1 and Sphk2) characterized by distinct cellular localization, tissue distribution, and physiological functions^5,7^. Although a large number of studies have focused on the role of Sphk1 in insulin resistance and lipid metabolism, the results are controversial. For example, Wang et al. found that Sphk1 deficiency could alleviate inflammation in adipose tissue and improve insulin sensitivity and glucose homeostasis in diet-induced obese (DIO) mice^8^. By contrast, Sphk1 was reported to promote pancreatic β-cell survival and protect DIO mice against diabetes^9^. Therefore, the role of Sphk1 in these metabolic diseases has not been fully elucidated, and more research is needed for further understanding.

The catalytic activity of Sphk1 could be increased by various stimulating factors, such as tumor necrosis factor-α (TNFα) and phorbol esters^10^. Moreover, post-translational modifications of Sphk1 have a great impact on its function. Phosphorylation of Sphk1 induced its translocation from cytoplasm to plasma membrane, and was required for its role in oncogenic signaling^11^. Our previous data also demonstrated that Sphk1 can be acetylated in a conserved GK acetylation motif. Interestingly, substitution of the two lysine (K) residues in this acetylation motif with glutamine (Q), which mimics the acetylated form of Sphk1, could influence protein stability and regulate cell growth, size, and cell cycle progression^12^. However, the underlying biological functions of acetylated Sphk1 were unclear.

To determine whether acetylation of Sphk1 was implicated in regulating glucose and lipid metabolism, we developed, for the first time, mice carrying Q residues in the GK acetylation motif of Sphk1 (QSPHK1 mice), and body fat accumulation and glucolipid metabolism of mice on a high-fat diet (HFD) were compared with those of WT mice on the same diet. The results showed that QSPHK1 mice were resistant to HFD-induced obesity, hepatic steatosis, and the development of insulin resistance in addition to being able to maintain glucose homeostasis.

## Results

### Generation of QSPHK1 knock-in mice

**Figure 1:**
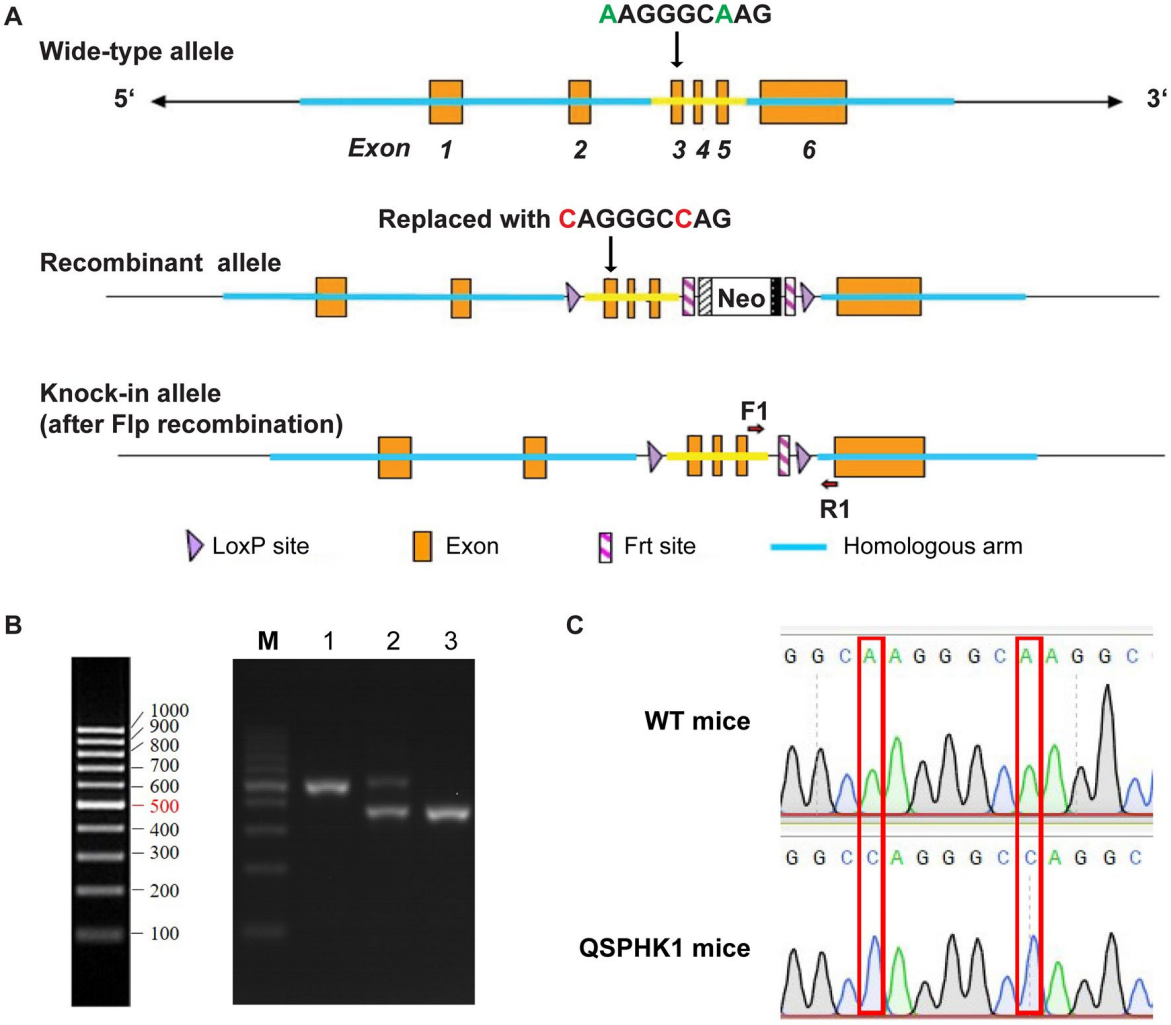
Generation of QSPHK1 knock-in mice. (**A**) Schematic diagram of Sphk1^K27Q K29Q^ (QSPHK1) targeting strategy. (**B**) Genotyping of QSPHK1 and WT mice. Genomic DNA was extracted from the tails of the mice and used as templates for PCR amplification of the region flanking the Frt site. Then PCR products were subjected to electrophoresis on a 1.5% agarose gel. Lane M: DNA marker. Lane 1 and 3: homozygous QSPHK1 and WT mice, respectively. Lane 2: heterozygous mice. (**C**) Confirmation of codon substitution of K27Q and K29Q in Sphk1 by sequencing. PCR products of exon 3 of S*phk1* were amplified with primers (SPKF: 5′- AGAGCAGCAAGTGCTCTTACCT-3′ and SPKR: 5′- GTCAGCACTCACCGGTGAGTAT -3′) and then sequenced.

The QSPHK1 knock-in mice were generated by gene targeting technology (Fig. 1A). The amino acid residues for lysines 27 (AAG) and lysines 29 (AAG) in exon 3 of Sphk1 were substituted with glutamine (CAG) on a C57BL/6 background (Sphk1^K27Q K29Q^). To generate the targeted construct, the 5′ homology arm and 3′ homology arm were amplified from BAC DNA and confirmed by end sequencing. The targeted construct contained a neomycin (Neo) cassette flanked by Frt sites in an intron and were transfected into ES cells from a C57BL/6 strain. The ES cells carrying the correct homologous recombination events were screened and confirmed by Southern analysis. The Neo cassette was then excised after Flp-mediated recombination, leaving a single Frt site. The Sphk1^K27Q K29Q^ (QSPHK1) knock-in mice were genotyped using PCR primers that flanked the Frt site (F1: 5′-GACTGGGAGACTGCCATCCAGAA-3′ and R1: 5′-CAGGTCTTCATTAGTCACCTGCTCG-3′), corresponding to a 361 bp product for the wild-type allele (WT) and a 489 bp product for the knock-in allele (Fig. 1B). Finally, both codon K27 and K29 were successfully substituted with Q without introducing other mutations in this region in the homozygous QSPHK1 knock-in mice as confirmed using genomic DNA sequencing (Fig. 1C).

The substitution of K27Q and K29Q in Sphk1 did not significantly alter the life span of the mice (Figure S1A). Moreover, the litter of QSPHK1 mice also showed similar size and sex ratios as for the WT mice (Figure S1B).

### QSPHK1 mice gained less body weight on a high-fat diet

**Figure 2:**
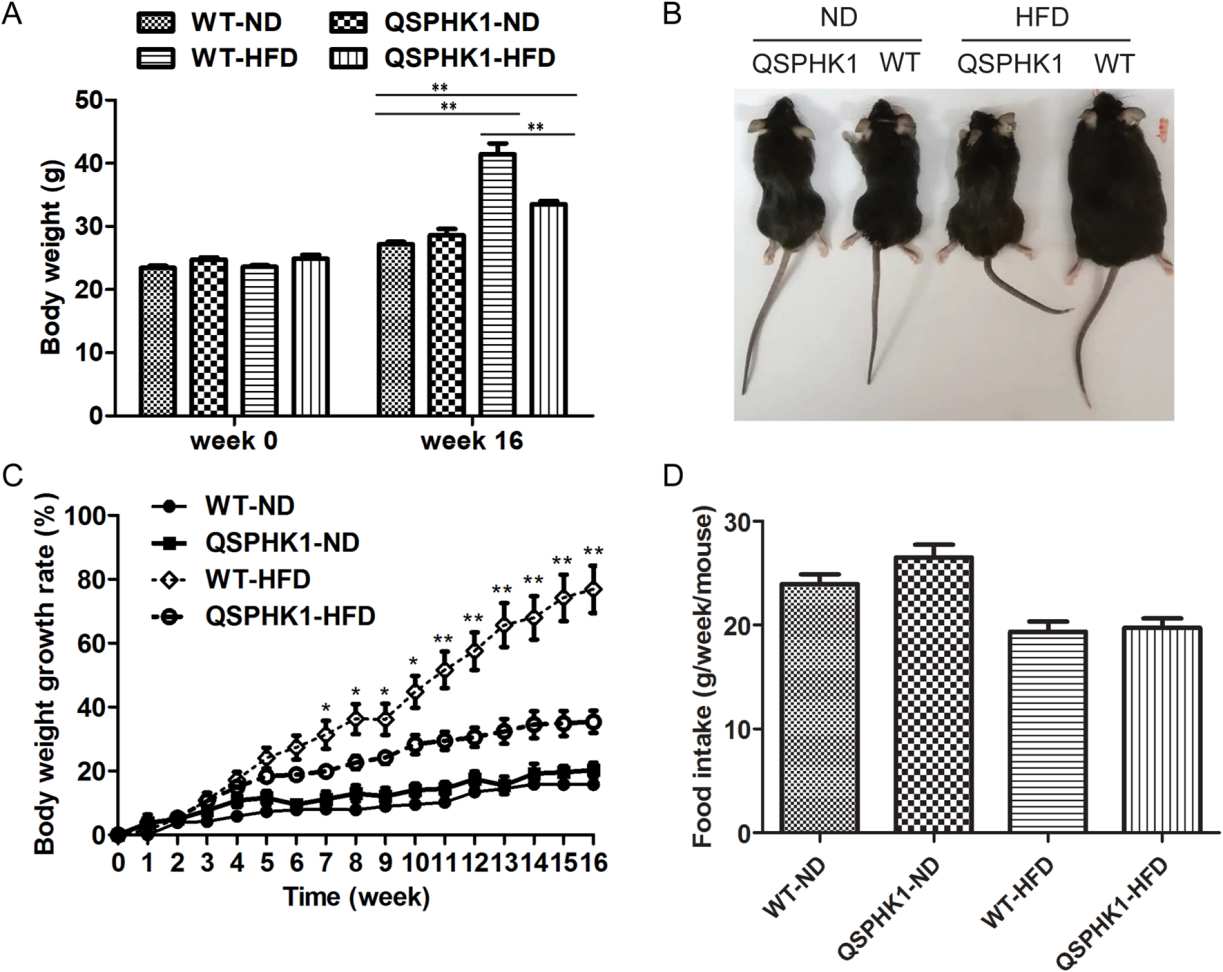
QSPHK1 knock-in mice showed reduced growth of body weight on a high-fat diet. (**A**) Body weight of WT and QSPHK1 mice on week 0 and after 16 weeks of either ND or HFD feeding. (**B**) Photos of representative mice in each group at the end of the experiment. (**C**) The growth of body weight of WT and QSPHK1 mice on either ND or HFD from 0 to 16 weeks. (**D**) Comparison of food intake between WT and QSPHK1 mice on either ND or HFD. Food intake was monitored once a week for 16 weeks and was expressed as grams per week per mouse. All data are presented as the mean ± SEM (n = 7 per group). *P value < 0.05, **P value < 0.01.

To study the influence of QSPHK1 knock-in mice on obesity, we fed two strains of mice a normal diet (ND) and high-fat diet (HFD), respectively. After 16 weeks, QSPHK1 mice gained comparable weight with WT mice on ND, but both strains were significantly obese when fed a high-fat food compared to normal diet (Fig. 2A). Surprisingly, QSPHK1 mice were obviously less obese than WT mice on HFD (Fig. 2A,B). The weight gain of WT mice on HFD was more rapid than for QSPHK1 mice, which became significantly different on week 7 and this difference continued to become more significant thereafter (Fig. 2C). Food intake was monitored throughout the experiment. However, no significant difference was observed between the food intake of QSPHK1 and WT mice, regardless of whether they were on ND nor HFD (Fig. 2D), indicating that the lower body weight gain of QSPHK1 mice was not due to reduced food intake.

### QSPHK1 mice reduced adiposity and improved serum lipid metabolism

**Figure 3:**
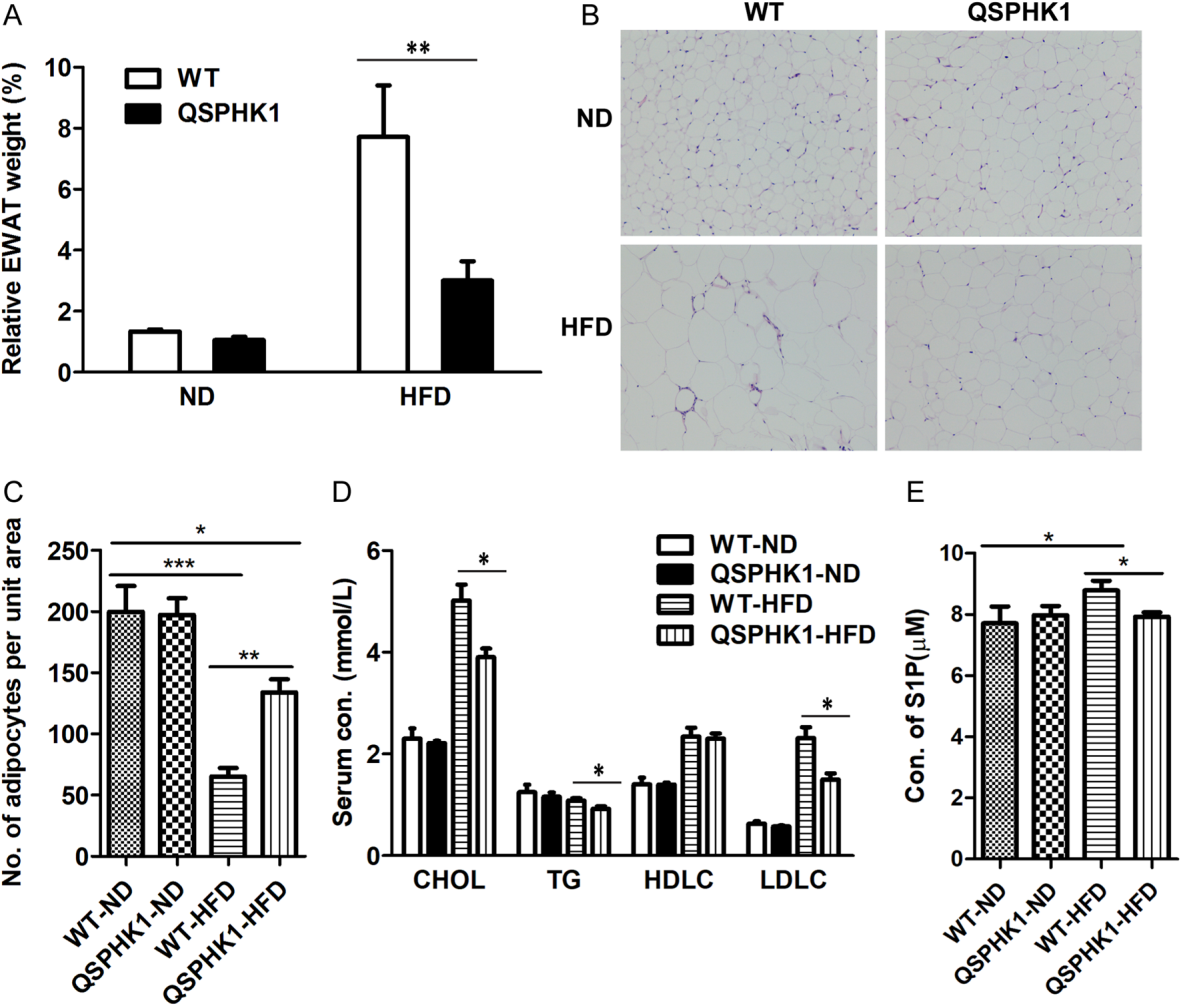
QSPHK1 knock-in mice accumulated less adipose tissue and improved serum lipid profile. (**A**) Relative weight of EWAT of WT and QSPHK1 mice fed with ND and HFD for 16 weeks. Data were expressed as EWAT mass to body weight ration (n = 7 per group). (**B**) Representative images of H&E staining of EWAT. (**C**) No. of adipocytes per unit area in EWAT counted from images of H&E staining (n = 4 per group). (**D**) Serum concentration of CHOL, TG, HDLC, and LDLC of mice in each group (n = 7 per group). (**E**) Serum concentration of S1P of mice in each group (N = 6–7 per group). Data are presented as the mean ± SEM. *P value < 0.05, **P value < 0.01, ***P value < 0.001.

At the end of the assay, the epididymal white adipose tissue (EWAT) of mice from each group was collected and weighted. There was no difference in the accumulation of EWAT between WT and QSPHK1 mice fed ND, whereas QSPHK1 mice accumulated remarkably less EWAT than WT mice fed HFD (Fig. 3A). Consistently, HFD feeding led to larger adipocytes than ND feeding. However, the adipocytes were apparently more enlarged in WT than in QSPHK1 mice fed HFD, whereas no adipocyte sizes differences were observed between the two strains of mouse fed ND, as shown in the EWAT sections (Fig. 3B,C).

To study the effect of QSPHK1 on serum lipid metabolism, the serum lipid contents in the serum of the four groups were assessed. Overall, WT and QSPHK1 mice fed ND showed comparable levels of serum parameters, but concentrations of cholesterol (CHOL), triglyceride (TG), and low-density lipoprotein cholesterol (LDLC) in the QSPHK1 mice fed HFD were significantly decreased compared to those of WT mice (Fig. 3D). A previous study showed that both obese mice and humans had elevated circulating S1P levels^13^. Consistent with this result, we found that serum S1P concentration was increased in WT mice fed HFD than mice fed ND (Fig. 3E). Interestingly, QSPHK1 mice fed HFD had comparable S1P levels with both WT and QSPHK1 mice fed ND (Fig. 3E). These results indicate that QSPHK1 mice had a higher capacity for lipid metabolisms.

### QSPHK1 mice reduced inflammation on HFD

**Figure 4:**
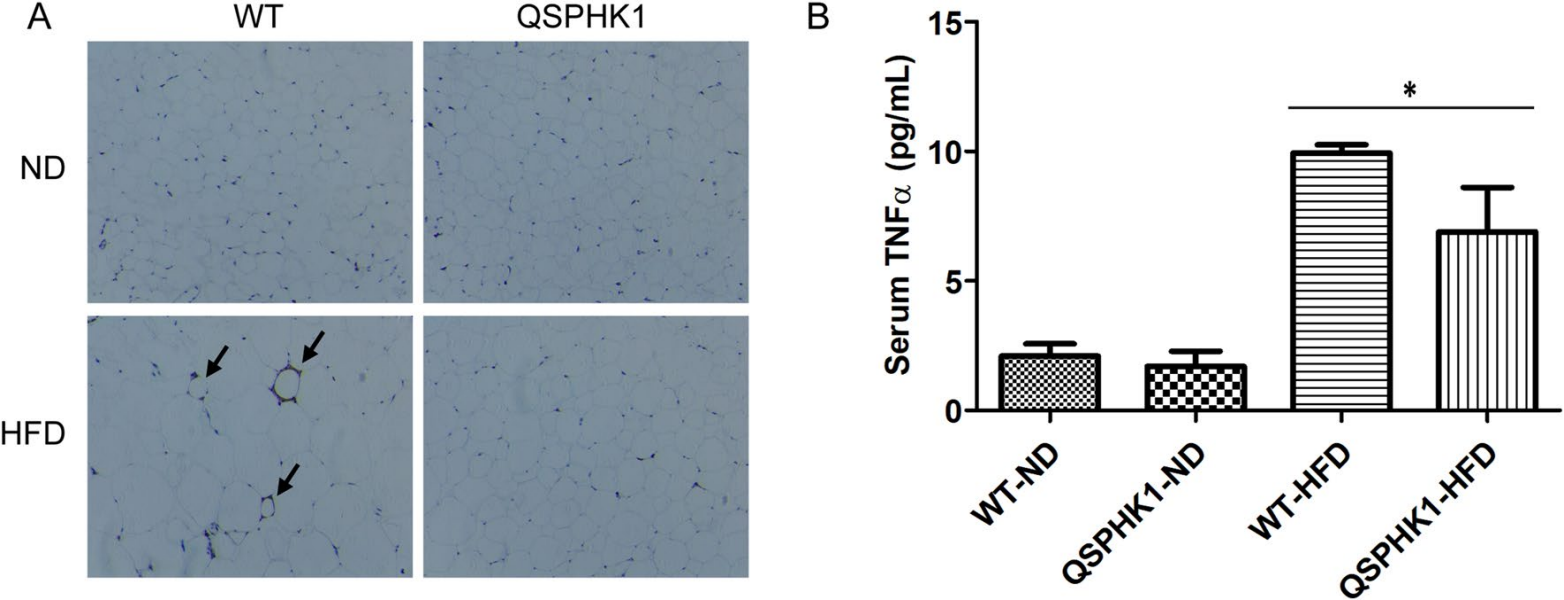
QSPHK1 mice reduced high-fat diet induced inflammation. (**A**) Representative images of F4/80 immunohistochemical staining of EWAT of WT and QSPHK1 mice fed with ND and HFD for 16 weeks (n = 4 per group). Arrows indicate positively stained cells. (**B**) Serum concentration of TNFα from mice in each group (n = 6–7 per group). Data are presented as the mean ± SEM. *P value < 0.05.

Since inflammation was reported to be closely related to obesity^14^, we detected macrophage infiltration into EWAT using immunohistochemical staining of the specific marker F4/80. As shown in Fig. 4A, F4/80-positive cells were prevalently observed in EWAT sections of WT mice fed HFD, forming a typical “crown-like” structure and indicating filtration of macrophages into the adipose tissue. By contrast, F4/80-positive cells were almost absent in QSPHK1 mice fed HFD, whose morphology was more similar to mice fed ND, indicating a limited inflammatory response in the adipose tissue of these mice.

We then assessed the systematic inflammation through the detection of proinflammatory factor TNFα in the sera, which plays a key role in obesity-induced insulin resistance. Consistent with previous reports, HFD feeding increased serum TNFα levels compared to ND feeding. However, the increase in TNFα of QSPHK1 mice was significantly less than for WT mice on HFD, suggesting that inflammation was alleviated in QSPHK1 mice (Fig. 4B).

### QSPHK1 mice alleviated high-fat-diet-induced hepatic steatosis

**Figure 5:**
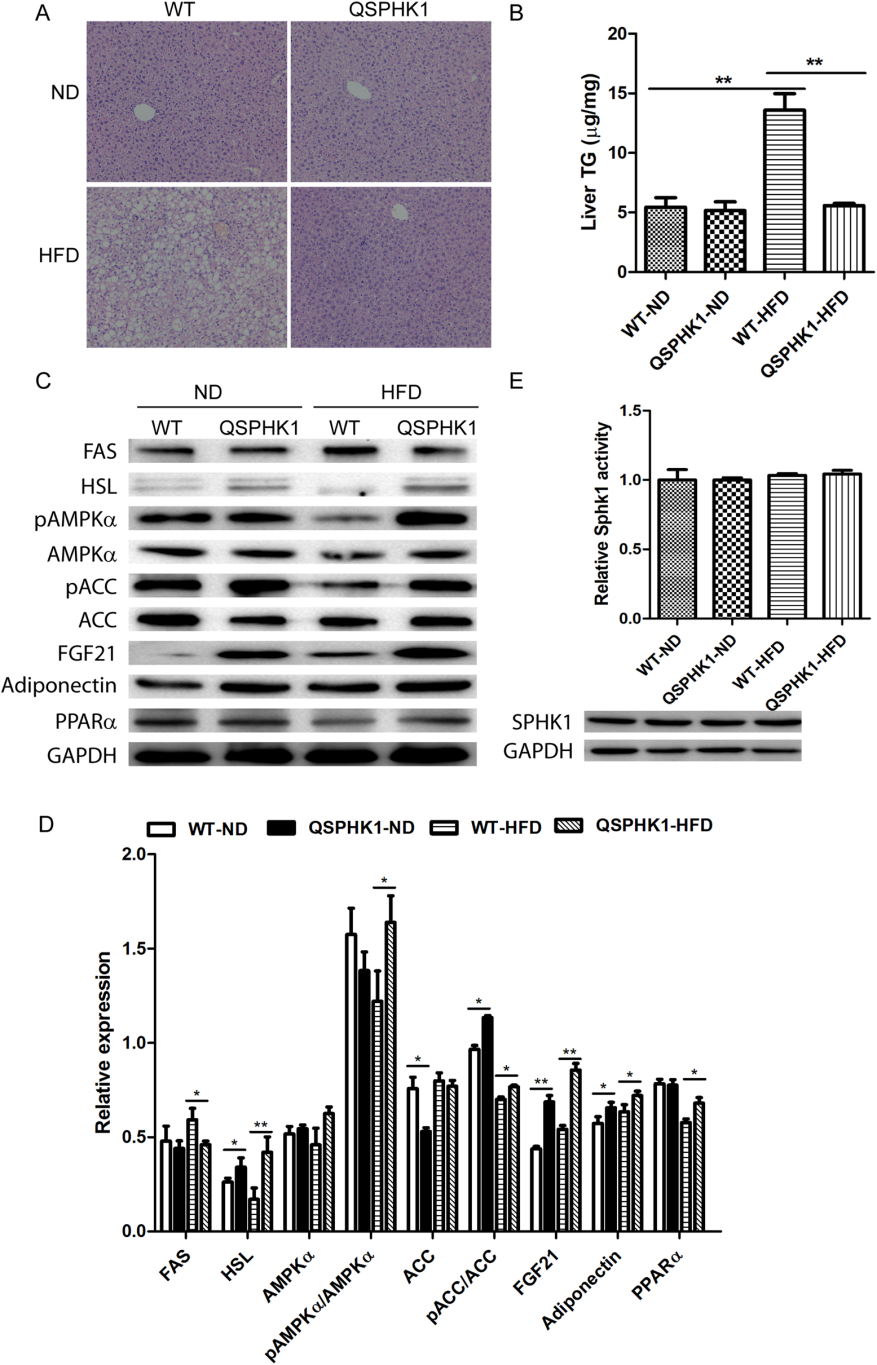
QSPHK1 mice prevented the development of HFD-induced fatty liver. (**A**) Representative images of H&E staining of liver tissues from WT and QSPHK1 mice fed with ND and HFD for 16 weeks (n = 4 per group). (**B**) TG content measured from liver extracts from mice in each group (n = 5 per group). (**C**) Immunoblotting analysis of proteins associated with lipid metabolism using liver tissue extracts. (**D**) Quantitative analysis of relative expression of genes detected in (**C**). Samples from three mice in each group were analyzed. The expression level of each protein was normalized to the reference gene GAPDH. Data are presented as the mean ± SEM. *P value < 0.05, **P value < 0.01. (**E**) The relative enzyme activity (upper) (n = 7 per group) and protein expression (down) of Sphk1 in the liver tissue (n = 3 per group). The enzyme activity of each group was normalized to the WT-ND group.

Because HFD contributes to the development of hepatic steatosis^15^, we evaluated the effect of QSPHK1 expression on HFD-induced hepatic steatosis through comparison of HE-stained liver sections from the four experimental groups. WT and QSPHK1 mice fed ND showed similar morphological structures of hepatic sections in which hepatocytes were compactly and orderly arranged. However, WT mice fed HFD displayed abnormal structures with obvious large lipid-droplet vacuoles in hepatocytes, indicating severe fatty denaturalization. Interestingly, the structure of hepatic sections of QSPHK1 mice fed HFD closely resembled that of mice fed ND in their lack of obvious fatty denaturalization (Fig. 5A).

The striking differences in hepatic microstructures of the four groups were further confirmed by analyzing the TG content of liver tissues. As shown in Fig. 5B, hepatic TG content was low in WT and QSPHK1 mice fed ND but increased dramatically in WT mice fed HFD. Consistent with H&E staining results, QSPHK1 mice fed HFD had a comparably low level of hepatic TG in comparison to mice fed ND, which indicated QSPHK1 mice accumulated less TG in hepatocytes and were more resistant to HFD-induced hepatic steatosis.

### QSPHK1 mice showed differences in hepatic lipid metabolism compared to WT mice

To dissect the mechanisms of reduced accumulation of hepatic lipids in QSPHK1 mice, we analyzed hepatic lipid metabolism by comparing the expression of several associated genes in liver extracts from the different experimental groups (Fig. 5C,D). As expected, the expression of a fatty acid synthesis gene, FAS, in WT mice fed HFD was significantly increased compared to QSPHK1 mice, whereas the expression of the lipolysis gene, HSL, was remarkably decreased in WT mice. Interestingly, the QSPHK1 mice fed ND also showed increased expression of HSL compared to WT mice. This indicates that the QSPHK1 mice had decreased fatty acid synthesis but increased fatty acid degradation, which is in accordance with the observed phenotypes.

AMPK is a key regulator of energy hemostasis, phosphorylating a variety of substrates involved in cellular metabolism, and is thus closely associated with insulin resistance, hepatic steatosis, and fatty acid metabolism^16^. Therefore, we characterized the expression of AMPKα and its activation by phosphorylation in hepatic samples. The results showed that the total level of AMPKα expression was the same in WT and QSPHK1 mice fed either ND or HFD, but QSPHK1 mice had increased levels of phosphorylated AMPKα /AMPKα compared to WT mice fed HFD. This suggests an increased activation of AMPK in the liver of QSPHK1 mice fed HFD, which is beneficial for the mice in reducing the accumulation of lipids in the liver and helping to maintain energy balance. ACC plays important roles in both fatty acid synthesis and oxidation pathways^17^. The phosphorylation of ACC by AMPK lead to ACC inactivation and, thus, prevented fatty acid synthesis and increased the fatty acid oxidation^16^. Using immunoblotting analysis, we found that in WT mice on ND, the total level of ACC expression was higher but that pACC/ACC was lower. Similarly, higher levels of pACC/ACC were detected in QSPHK1 mice on HFD although the total level of ACC expression was comparable to that of WT mice. These results implicate the AMPK/ACC axis in the prevention of HFD- induced hepatic steatosis of QSPHK1 mice.

Several lines of evidence have indicated that FGF21 plays a central role in regulating glucose and lipid metabolism, as well as the maintenance of energy homeostasis^18,19^. Adiponectin is an adipokine that was found to be functionally linked with FGF21 and involved in the pathogenesis of metabolic disorders^20^. Intriguingly, we found both FGF21 and adiponectin had increased expression in the livers of QSPHK1 mice both on ND and HFD as compared with WT mice. Consistently, the expression of PPARα, a key activator of FGF21^21^, was also upregulated in QSPHK1 mice on HFD. This indicates that FGF21 and adiponectin also mediate the accumulation of hepatic lipids in QSPHK1 mice.

Next, we investigated whether the differential expression of various metabolic related genes between QSPHK1 and WT mice were connected to the distinct hepatic expression and enzyme activity of Sphk1. However, similar expression levels and relative activity of Sphk1 were observed between the two mouse strains, which was independent of diet (Fig. 5E). This indicates that the variations in hepatic morphologies and lipid metabolism between QSPHK1 and WT were not due to changes in expression or enzyme activity.

### QSPHK1 mice on HFD maintain glucose homeostasis and have ameliorated insulin resistance

**Figure 6:**
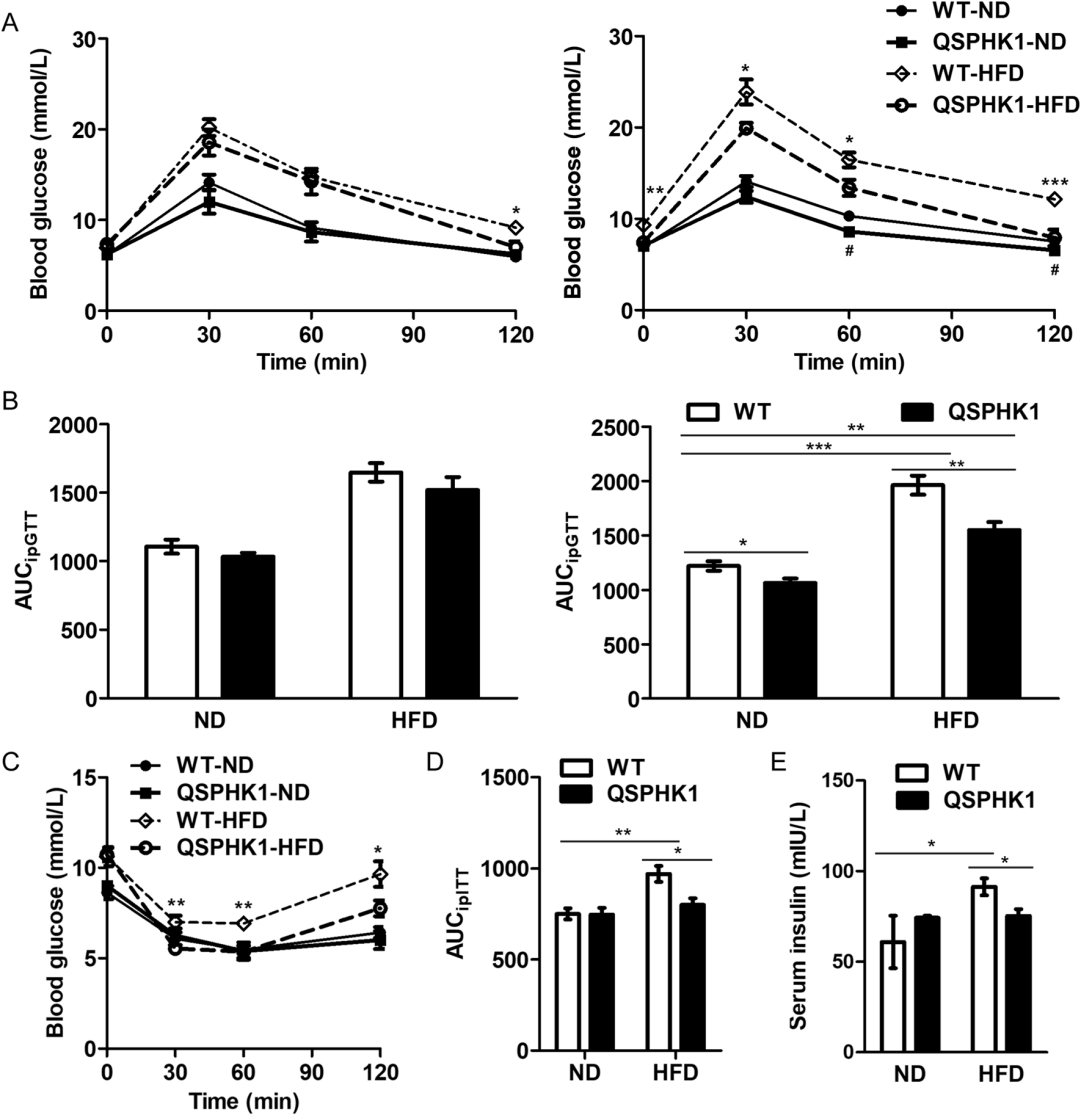
Glucose metabolism and insulin tolerance were improved in QSPHK1 mice. (**A**) Intraperitoneal glucose tolerance test (ipGTT) in WT and QSPHK1 mice fed with ND and HFD for 8 weeks (left) and 16 weeks (right), respectively. (**B**) The area under the curves (AUCs) of blood glucose measured from ipGTT results for 8 weeks (left) and 16 weeks (right), respectively. (**C**) Intraperitoneal insulin tolerance test (ipITT) in WT and QSPHK1 mice fed with ND and HFD for 17 weeks and the corresponding AUC (D). (E) The concentration of serum insulin in each group. Data are presented as the mean ± SEM (n = 7 per group). *P value < 0.05, **P value < 0.01, ***P value < 0.001.

To determine the role of QSPHK1 in glucose metabolism, ipGTT was performed at week 8 and 16. At week 8, there were no significant differences in blood glucose levels between WT and QSPHK1 mice fed ND at the four tested time points, nor in their AUCs (Fig. 6A,B). Although WT and QSPHK1 mice fed HFD showed the same levels of glucose at 0, 30, and 60 min following the injection of insulin, WT mice had significantly higher glucose at 120 min versus QSPHK1 mice (Fig. 6A). The AUC_ipGTT_ values for WT versus QSPHK1 mice on HFD were comparable at week 8 (Fig. 6B). This indicates that QSPHK1 mice have slight advantages regarding glucose metabolism after 8 weeks of feeding. Interestingly, WT and QSPHK1 mice showed great differences in ipGTT for both ND and HFD (Fig. 6A,B) at week 16. On ND, the glucose levels of QSPHK1 mice were lower than those of WT mice at 60 and 120 min following the injection of insulin, and the AUC_ipGTT_ of QSPHK1 mice was less than WT mice. When fed with HFD for 16 weeks, the glucose of WT mice was significantly higher than QSPHK1 mice at each timepoint, including for fasting glucose (0 min). The AUC_ipGTT_ for week 16 also showed the same tendency. These results indicate that QSPHK1 mice demonstrate superiority in the maintenance of glucose homeostasis with age, which was independent of diet.

To further study whether QSPHK1 influences insulin sensitivity, ipITT were performed at week 17. As expected, HFD feeding of WT mice resulted in apparent insulin resistance compared to ND feeding. For ipITT, QSPHK1 mice fed HFD showed obviously lower levels of glucose than WT mice at 30, 60, and 120 min after injection of insulin. The results for AUC_ipITT_ were consistent. Surprisingly, the glucose level of QSPHK1 mice fed HFD was almost the same as their littermates fed ND, as shown in the ipITT results (Fig. 6C,D). The serum insulin level was also measured, and it showed that WT mice fed HFD had significantly increased insulin than their littermates fed ND as well as compared to QSPHK1 mice fed HFD. However, there was no difference in serum insulin between QSPHK1 mice fed HFD and ND (Fig. 6E). This suggests that QSPHK1 mice had improved insulin sensitivity on HFD.

## Discussion

S1P and ceramide (Cer) are two key intermediates in sphingolipid metabolism, which are critical in determining cell fate and regulating several biological activities including immune cell trafficking, glucose homeostasis, and lipid metabolism^6,22^. The enzyme Sphk1 catalyzes phosphorylation of Sph, the metabolite of ceramide, to form S1P, and thus controls the balance of Cer/Sph and S1P, which is known as the ‘sphingolipid rheostat’^23^. Therefore, Sphk1 are implicated in several metabolic diseases, such as obesity, diabetes, and cardiovascular disease^24^. In this study, we used genetically modified mice that constitutively expressed Sphk1^K27Q K29Q^ (QSHPK1) to study its role in regulating obesity and the associated metabolic disorders. Interestingly, we found QSHPK1 mice were better in controlling HFD-induced obesity, hepatic steatosis, and inflammation. Moreover, QSHPK1 mice showed advantages against insulin resistance and maintaining glucose homeostasis. The results suggest that the K27Q/K29Q mutations of Sphk1 could serve a beneficial role in regulating lipid and glucose metabolism and thereby protect mice from obesity and its associated pathologies.

Point mutations could lead to gain-of-function and loss-of-function of several metabolic genes, such as proprotein convertase subtilisin/kexin type 9 (*PCSK9*) and patatin-like phospholipid domain containing protein 3 (*PNPLA3*)^25,26^. Indeed, the effects of point mutations of Sphk1 on its functions have also been extensively investigated. For example, G113A mutagenesis of human Sphk1 could directly increase the activity of the enzyme^10^. However, human Sphk1^S225A^ influenced its phosphorylation and translocation without altering the expression and enzyme activity^11^. In this study, we described the mutations of K27Q/K29Q of mouse Sphk1, which was located at the N terminal of the conserved DAG catalytic domain^12^. We showed that this mutagenesis of Sphk1 in mice had a dramatic impact on the glucolipid metabolism that was independent of the expression and enzyme activity as detected in the liver (Fig. 5E). Sphk1 could be located at the cytoplasm, plasma membrane, or exported extracellularly to play a key role in sphingolipid metabolism^27,28^. However, little is still known about the molecular mechanisms and role of extracellular export of Sphk1. To date, three mouse Sphk1 (mSphk1) isoforms due to alternative splicing have been identified: mSphk1a, mSphk1a2, mSphk1b^29^. These splice variants, which mainly differed at the N-terminus close to our knock-in site, were different in cellular location, stability, post-translational modification^30^. Therefore, it would be quite interesting to study, in the future, whether K27Q/K29Q mutations in Sphk1 could change Sphk1 splice variant expression and influence extracellular secretion of this enzyme, and thus leading to the different phenotypes of the QSHPK1 and WT mice in this study.

It has been widely accepted that obesity is a risk factor for hepatic steatosis, characterized by overload of TG in the liver^15^. Accordingly, we showed that HFD induced obvious accumulation of TG and severe steatosis in the liver of WT mice. However, HFD played a limited role in the development of hepatic steatosis in QSPHK1 mice, which showed similar phenotypes as mice fed ND (Fig. 5). An imbalance between fatty acid synthesis and disposal in the liver would lead to steatosis^15^. Although obese WT mice had increased expression of FAS and decreased expression of HSL in the liver, QSPHK1 HFD mice showed comparable levels of FAS expression as ND mice and remarkably increased HSL expression. This suggests that QSPHK1 mice are more capable of disposing dietary fat and combatting hepatic steatosis. Further studies showed that the AMPK/ACC and FGF21/adiponectin axes appear to be implicated in the processing of dietary fat in QSPHK1 mice. The regulation of AMPK/ACC in inducing hepatic lipid oxidation and inhibiting de novo lipogenesis has been reported in other papers^17^. In our work, the increased activation of the AMPK/ACC pathway was observed in the liver of QSPHK1 mice. We also detected an elevation of FGF21 and adiponectin expression in the liver of the QSPHK1 mice, which favors fat oxidation and suppressed lipogenesis^31^. Moreover, the role of FGF21 in lipid and energy metabolism is closely associated with activation of AMPK^18^. Therefore, there could be crosstalk between the AMPK/ACC axis and the FGF21/adiponectin axis in the regulation of hepatic fatty acid metabolism in QSPHK1 mice.

Inflammation has been closely linked with obesity-induced insulin resistance^2^. In obesity, the recruitment of adipose tissue macrophages (ATMs) is an early event of tissue inflammation. The activated ATMs then secrete various proinflammatory cytokines, such as TNF-α and interleukin-1β (IL-1β), which could lead to systemic insulin resistance due to endocrine actions^32^. Consistently in this study, HFD feeding was observed to induce obesity and increased serum TNFα levels, and apparent infiltration of ATMs were observed in WT HFD mice, which also showed obvious characteristics of insulin resistance, i.e., increased fasting insulin levels, postprandial hyperglycemia, impaired glucose tolerance, and insulin tolerance (Figs. 4 and 6). However, QSHPK1 HFD mice showed a decreased serum TNFα levels and ATM infiltration, as well as less severe insulin resistance. Among several inflammatory signaling pathways associated with obesity induced insulin resistance, TNFα signaling was induced by tumor necrosis factor-α receptor-associated factor 2 (TRAF2), activating JNK and IKK, which contributed to the development of insulin resistance^32^. Interestingly, Sphk1 was found to directly bind to TRAF2 and influence downstream signaling^33^. A recent study demonstrated that Sphk1 could promote the survival of ATMs under lipotoxic conditions^34^. Therefore, it can be inferred that QSPHK1 might inhibit the survival and function of ATMs, thus reducing secretion of proinflammatory cytokines, or acting downstream by interfering with TNFα /TRAF2 signaling pathways. However, further studies are needed to dissect the suppressive effects of QSPHK1 on inflammation and obesity induced insulin resistance.

In conclusion, our work shows that Sphk1 plays a critical role in glucose, lipid, and energy metabolism. The K27Q/K29Q mutations of Sphk1 in mice resulted in amelioration of HFD-induced obesity. Improved insulin sensitivity and reduced hepatic steatosis were observed in HFD-induced QSPHK1 mice. The benefits conferred bySphk1^K27QK29Q^ in preventing deterioration due to metabolic disorders, uncovered in this study, provide new insights into the development of strategies to treat metabolic diseases, including obesity, diabetes, and NAFLD.

## Experimental procedures

### Animals

All experiments were performed with homozygous QSPHK1 knock-in mice and their WT littermates. All experimental procedures were approved and in accord with the Animal Care and Use Committee of Academy of Military Medical Sciences, China. Male WT and QSPHK1 mice were fed with palmitate-rich high-fat diet (HFD; 60% kcal from fat, D12492; Research Diets) or normal diet (ND) beginning at 8-week of age (defined as week 0). Food and drinking water were supplied ad libitum. All mice were maintained on a 12/12-h light/dark cycle. Body weights and food intake were monitored weekly. Intraperitoneal glucose tolerance test (ipGTT) was performed at week 8 and 16, respectively. Intraperitoneal insulin tolerance test (ipITT) was performed at week 17. Subsequently, mice were sacrificed by cervical dislocation.

### Intraperitoneal glucose tolerance test (ipGTT)

Mice were fasted for 10 h until the glucose tolerance test was performed. Glucose was intraperitoneally administered at a dose of 1 g/kg body weight. Glucose was measured by the glucocard test (Roche) from blood collected from the tail vein at 0, 30, 60 and 120 min, respectively.

### Intraperitoneal insulin tolerance test (ipITT)

For ipITT, mice were fasted for 6 h and then insulin were intraperitoneally administered at a dose of 0.7 U/kg body weight. Blood were collected and glucose were measured as ipGTT.

### Histological and immunohistochemical analysis

Epididymal white adipose tissue (EWAT) and liver tissues were fixed in 4% paraformaldehyde at room temperature for at least 24 h, and then embedded in paraffin wax. 5 mm-sections were cut and stained with haematoxylin and eosin. Immunohistochemistry of F4/80 positive macrophages in EWAT sections was performed as described elsewhere^35^.

### Analysis of serum content and hepatic TG content

Blood was collected from mice eyes before cervical dislocation. Serum was separated after centrifuging at 4000*g* for 20 min, and samples were stored at -80 °C for further analysis. The detection of serum CHOL, TG, HDLC, LDLC and insulin levels was performed as previously described^36^. Serum S1P level was determined using a sphingosine 1-phosphate assay kit (Echelon Biosciences Inc.) according to the manufacturer’s instructions.

For the measurement of hepatic TG content, 0.1 g tissue extracts from liver were analyzed using a triglyceride colorimetric assay kit following the manufacturer’s protocol (Cayman Chemical, Michigan, USA).

### ELISA analysis of TNFα

Serum TNFα level was measured by an enzyme-linked immunosorbent assay (ELISA) according to the manufacturer’s instructions (Neobioscience, Beijing, China).

### Western blot analysis

Total protein was extracted from the liver tissues with RIPA lyses buffer containing 1% proteinase inhibitor and 1% PMSF. 60 µg of protein samples was subjected to western blot analysis. Immunoblotting was performed using antibodies against fatty acid synthase (FAS), hormone-sensitive lipase (HSL), AMP-activated protein kinase (AMPKα), Phospho-AMPKα (Thr172), acetyl-CoA carboxylase (ACC), Phospho-ACC (Ser79) and adiponectin (purchased from Cell Signaling Technology), and fibroblast growth factor 21 (FGF21), peroxisome proliferator-activated receptorα (PPARα) antibody (purchased from abcam). Glyceraldehyde-3-phosphate dehydrogenase (GAPDH) was used as reference.

### Measurement of Sphk1 activity

0.1 g tissue extracts from liver were used for Sphk1 activity assay with a commercial kit according to the manufacturer’s instructions (Sphingosine Kinase Activity Assay Kit, Echelon Biosciences Inc.).

### Statistical analysis

Data are presented as mean ± SEM. Comparison was conducted using unpaired t-tests or one-way ANOVA with Graphpad Prism software. Significant differences were defined at the *P* < 0.05 level.

## Supplementary information

Supplementary Information 1.
