# Supplementary material for: K27Q/K29Q mutations in sphingosine kinase 1 attenuate high-fat diet induced obesity and altered glucose homeostasis in mice
Source: Sci Rep. 2020 Nov 18;10:20038. doi: 10.1038/s41598-020-77096-w (PMC7676274; doi:10.1038/s41598-020-77096-w)
Supplement: Supplementary file 1 — Supplementary Information 1. [file 41598_2020_77096_MOESM1_ESM.pdf]

Supplemental Information

K27Q/K29Q mutations in sphingosine kinase 1 attenuate high-fat diet induced obesity and altered glucose homeostasis in mice

Jing Xie[1,3], Yong Shao[2,3], Jin Liu[1], Meilan Cui[1], Xiuxiao Xiao[1], Jingbo Gong[1], Binghua Xue[1], Qunwei Zhang[1], Xianwen Hu[2,*], and Haifeng Duan[1,4,*]

Supplementary Figures

A

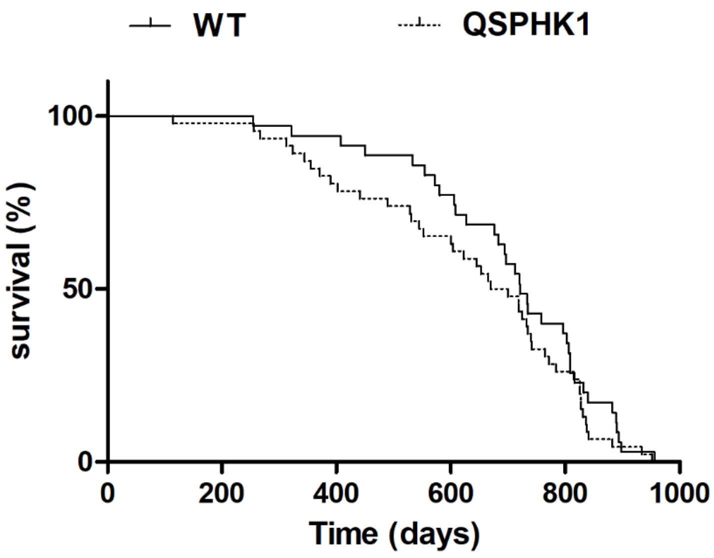

B

	Litter size (n)	Litter numbers	The ratio of male offspring
WT	6.62 ± 2.17	17 (n=162)	50%
QSPHK1	5.93 ± 2.24	21 (n=208)	56%

Figure S1. No differences in life span, litter size and sex ratio were observed between QSPHK1 knock-in mice and their wide-type littermates. (A) The survival curves of QSPHK1 knock-in mice and wide-type mice (WT). QSPHK1 (n=61) and WT (n=58) mice were fed with normal diet *ad libitum* until natural death. The P value is 0.2233. (B) Comparison of the litter size and the ratio of male offspring between QSPHK1 and WT mice. The P value is 0.3508.

Original blots for Figure 5C

**Target protein**	WT-ND, QSPHK1-ND, WT-HFD, QSPHK1-HFD	**Exposure time**
FAS	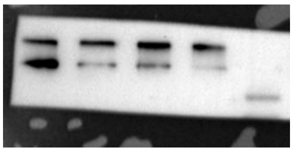	60s
FAS	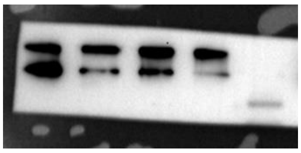	120s
HSL	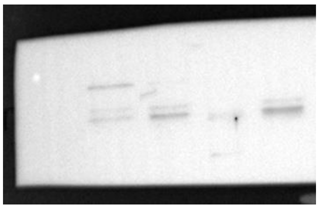	5min
HSL	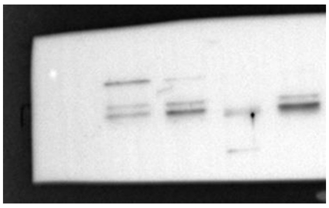	10min
pAMPKα	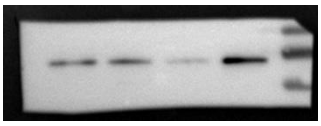	60s
pAMPKα	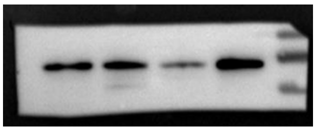	120s
AMPKα	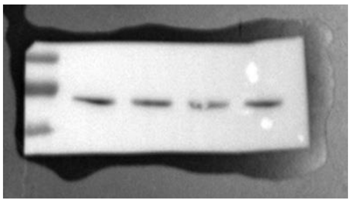	60s
AMPKα	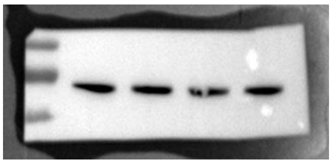	120s

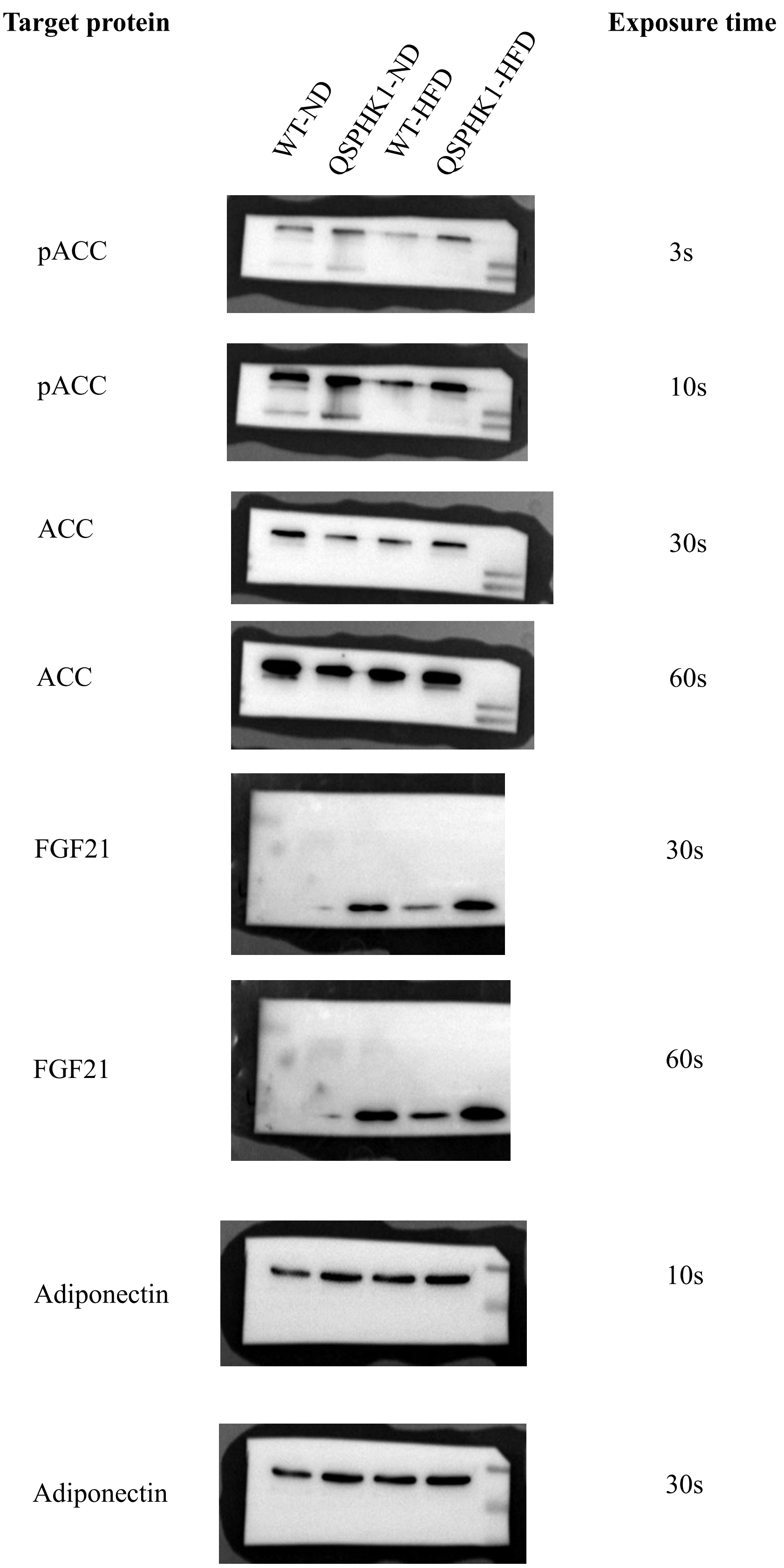
Target protein
Exposure time
WT-ND
QSPHK1-ND
WT-HFD
QSPHK1-HFD
pACC
3s
pACC
10s
ACC
30s
ACC
60s
FGF21
30s
FGF21
60s
Adiponectin
10s
Adiponectin
30s

WT-ND QSPHK1-ND WT-HFD QSPHK1-HFD

Target protein		Exposure time
PPARα	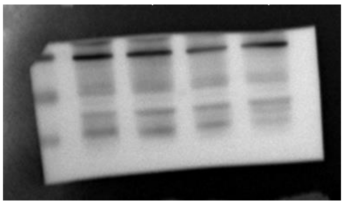	10s
PPARα	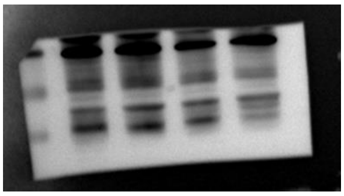	30s
GAPDH	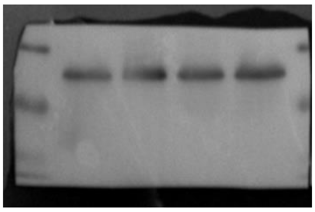	3s
GAPDH	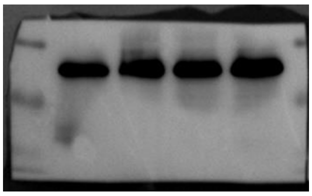	10s

Original blots for Figure 5E

Target protein	WT-ND, QSPHK1-ND, WT-HFD, QSPHK1-HFD	Exposure time
GAPDH<br>SPHK1	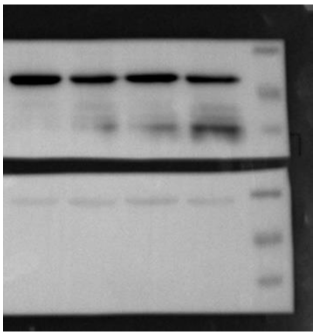	3s
GAPDH<br>SPHK1	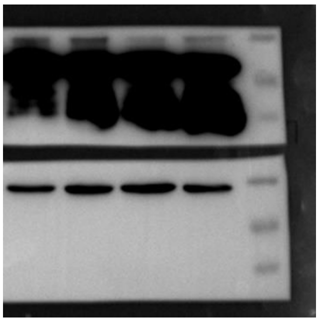	30s
